# Neurons of the Dentate Molecular Layer in the Rabbit Hippocampus

**DOI:** 10.1371/journal.pone.0048470

**Published:** 2012-11-07

**Authors:** Francisco J. Sancho-Bielsa, Juan D. Navarro-López, Gregori Alonso-Llosa, Asunción Molowny, Xavier Ponsoda, Javier Yajeya, Carlos López-García

**Affiliations:** 1 Cellular Neurobiology, Department of Cell Biology, University of Valencia, Valencia, Spain; 2 Laboratory of Neurophysiology and Behaviour, School of Medicine of Ciudad Real, University of Castilla-La Mancha, Ciudad Real, Spain; 3 Department of Physiology and Pharmacology, University of Salamanca, Salamanca, Spain; IRB Barcelona, Parc Cientific de Barcelona and CIBERNED (ISCIII), University of Barcelona, Spain

## Abstract

The molecular layer of the dentate gyrus appears as the main entrance gate for information into the hippocampus, i.e., where the perforant path axons from the entorhinal cortex synapse onto the spines and dendrites of granule cells. A few dispersed neuronal somata appear intermingled in between and probably control the flow of information in this area. In rabbits, the number of neurons in the molecular layer increases in the first week of postnatal life and then stabilizes to appear permanent and heterogeneous over the individuals’ life span, including old animals. By means of Golgi impregnations, NADPH histochemistry, immunocytochemical stainings and intracellular labelings (lucifer yellow and biocytin injections), eight neuronal morphological types have been detected in the molecular layer of developing adult and old rabbits. Six of them appear as interneurons displaying smooth dendrites and GABA immunoreactivity: those here called as *globoid, vertical, small horizontal, large horizontal, inverted pyramidal* and *polymorphic*. Additionally there are two GABA negative types: the *sarmentous* and *ectopic granular neurons*. The distribution of the somata and dendritic trees of these neurons shows preferences for a definite sublayer of the molecular layer: small horizontal, sarmentous and inverted pyramidal neurons are preferably found in the outer third of the molecular layer; vertical, globoid and polymorph neurons locate the intermediate third, while large horizontal and ectopic granular neurons occupy the inner third or the juxtagranular molecular layer. Our results reveal substantial differences in the morphology and electrophysiological behaviour between each neuronal archetype in the dentate molecular layer, allowing us to propose a new classification for this neural population.

## Introduction

The main entrance gate of sensory information to the hippocampus is the molecular layer of the dentate gyrus. The arrangement of this layer is like a highly laminated structure that receives axons not only from the entorhinal cortex in their outer and intermediate strata (perforant pathway), but also from contralateral CA3 pyramidal axons, as well as hilar neurons in the inner stratum (contralateral pathway) and a few diffuse axonal projections from the septum and mesencephalic nuclei (locus ceruleus norepinephric, mammillary histaminergic and raphe serotonergic axons) [1].

The neuronal population of the molecular layer is very sparse. The first classical neuronal morphology description of their neurons was based on the rabbit hippocampus [2]. In this report, Ramón y Cajal described two groups of cells *“triangular cells or displaced granular”* and *“short axon cells”* which divide into two other groups, these being superficial and deep cells with several somatic morphologies. The next classical study [3] maintains the same classification and nomenclature in terms of the hippocampal molecular layer neuronal population, but this study was conducted on mice. No mention of these neurons was made in the bibliography until the study of the rabbit hippocampus histogenesis [4]–[7]. Later, descriptions of displaced basket cells [8], [9], or ectopic granular cells with vertical morphology [10], and *neurogliaform* cells [11] in the molecular layer of rats were provided.

**Figure 1:**
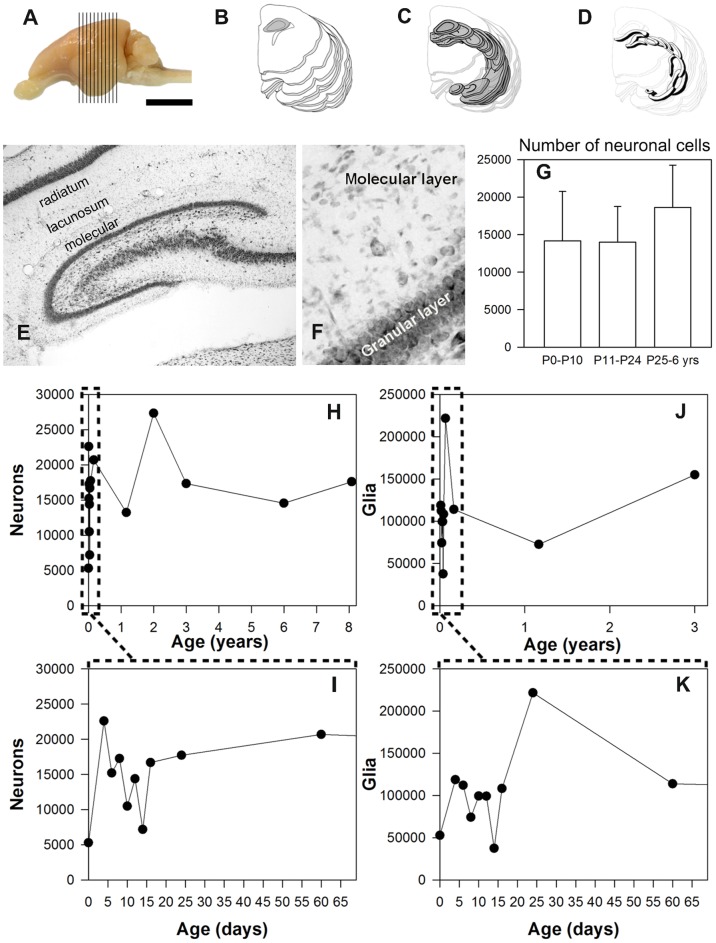
Hippocampal structure, nissl staining and cell counts along development. *A,* lateral view of the left hemisphere of a rabbit (P30), vertical lines show the levels of the transversal sections studied. *B,* Drawing of the silhouettes; profiles of the transversal sections, from anterior to posterior, dark gray marks the hippocampus. *C*, Drawing of transparent sections with the exception of the hippocampus. *D*, Drawing as only an opaque molecular layer. *E*, Nissl staining in a transversal section of a P12 rabbit showing the extent of the molecular layer. *F,* Enlarged view of the molecular layer with some neuronal somata and abundant glial cells. *G,* Histogram showing the estimated mean numbers of the neurons in the molecular layer of one hemisphere for postnatal, young and adult rabbits (vertical bars mean deviation). *H,* Plots of the estimated numbers of neurons in the molecular layer of one hemisphere after the stereological study. *I,* Enlarged view of the first two months of life. *J,* Plots of the numbers of glia. *K,* Enlarged view of the first two months of life.

Most cells in the molecular layer of the mouse hippocampus are called Cajal-Retzius cells and, in mice, it has been shown that many of them disappear in the third postnatal week coincidentally with the arrival of the entorhinal perforant axons [12], [13]. The remaining/surviving cells in the molecular layer of rats have been classified into four categories: stellate, pyramidal-like, displaced granular, and a new type which projects to the subiculum [14]. More recently, particular calretinin immuno-reactive neurons in the molecular layer of domestic pigs have been described [15], [16] as well as the presence of neurogliaform neurons in rats [11]. The postnatal development and functional maturation of this complex neuronal population in the dentate gyrus molecular layer has been also examined [17].

The dentate molecular layer is one of the first damaged or altered structures of several pathological processes such as Alzheimer’s disease, vascular fibrosis and calcification in the hippocampus, epilepsy, etc., and suggests that the neuronal population of this layer could play an important role for correct hippocampus operation. Despite the systematic studies of hippocampal interneurons [18]–[20], the study of the molecular layer has not been systematically carried out (neither the location nor the typology of its neuronal population). In the present work, we have identified eight different neuronal morphological archetypes with a particular laminar distribution in the molecular layer. Six of them appear as interneurons displaying smooth dendrites and GABA immunoreactivity whereas other two are GABA negative: those here called as sarmentous and some ectopic granular neurons. A brief electrophysiological approach has assessed differences between these morphological archetypes.

**Figure 2:**
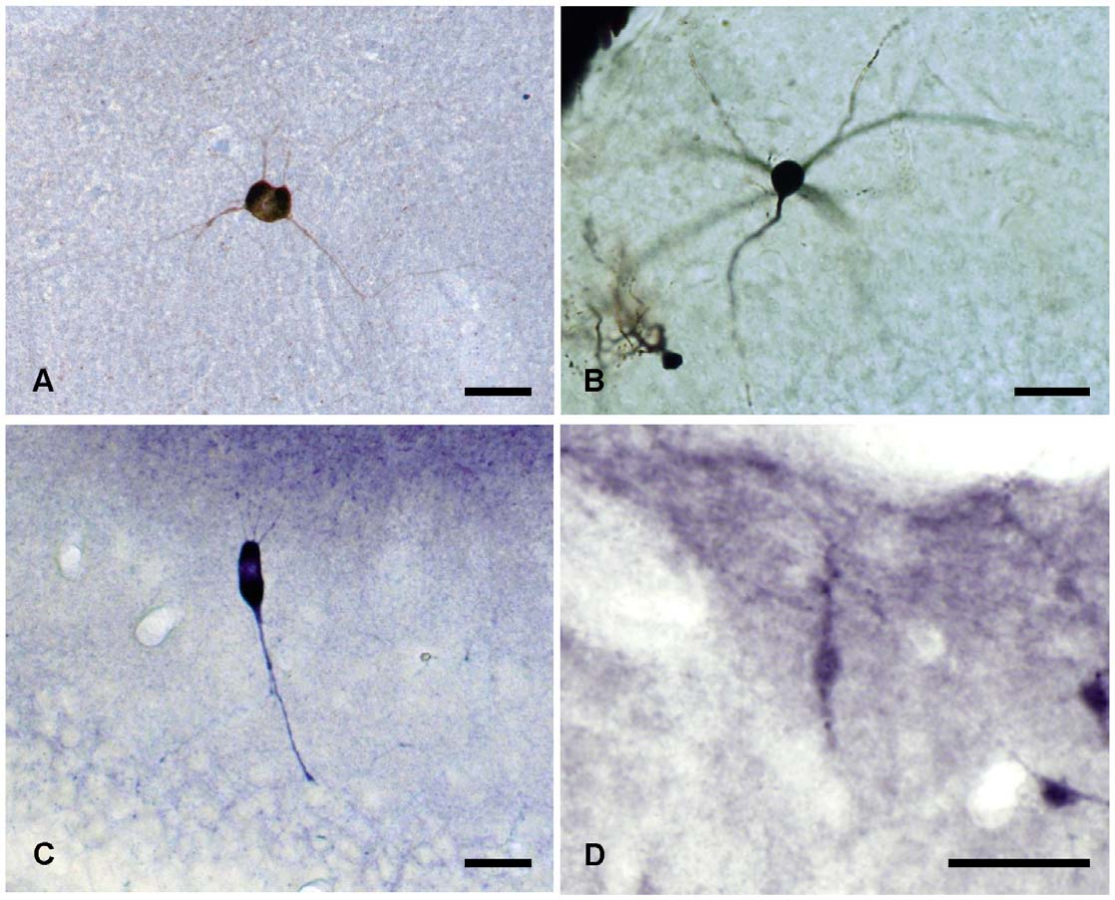
Stainings of globoid and vertical neurons. *A,* Calbindine immunostaining of a globoid soma with very thin dendrites, *B,* Golgi-impregnated globoid neuron, *C,* NADPH diaphorase histochemical staining of a vertical neuron, *D,* Calretinin immunostaining of a vertical neuron. Scale bars, *A–D*: 25 µm.

**Figure 3:**
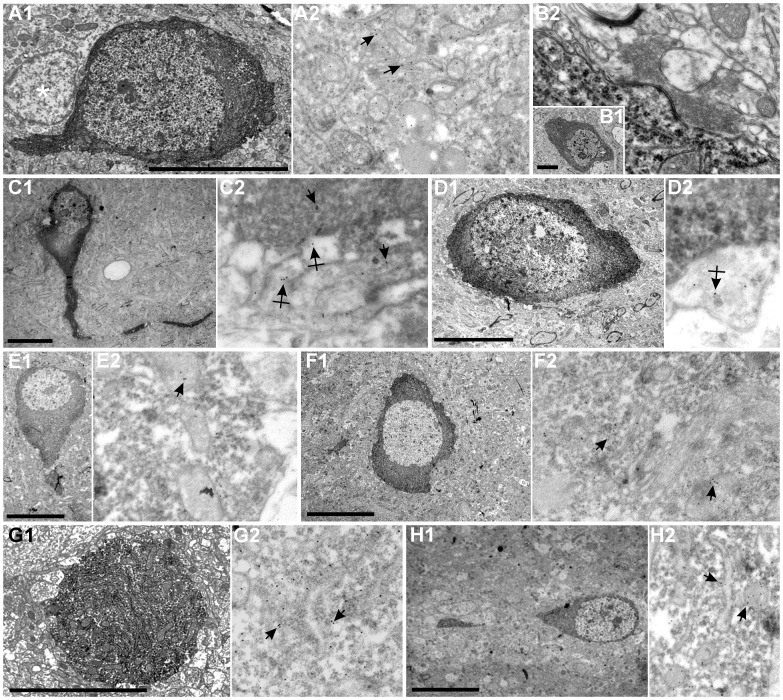
Electron micrographs of hippocampal molecular layer neurons. Different cellular archetypes were firstly injected with Lucifer Yellow and then filled with biocytin (see methods). In these stained neurons, GABA was detected using a post-embedding immunogold (immunoparticles 10 nm) method (arrows). *A1*: Biocytin-stained globoid neuron soma. *A2*, Soma magnification showing immunoparticles for GABA mainly detected in the soma. Asterisk shows a glial cell close to the injected neuron. *B1, B2*: Sarmentous neuron soma and magnification without immunoparticles. *C1, C2*: Vertical neuron and amplification of immunogold particles detected in the soma (arrows) and presynaptic contacts (crossed arrows). *D1, D2*: Ectopic granular neuron. Immunogold particles were detected in presynaptic contacts (crossed arrows) localized along somatic and dendritic plasma membrane. *E, F, G* and *H* micrographs show, respectively, inverted pyramidal, polymorphic, large horizontal and small horizontal neurons with immunoparticles magnification mainly located in the soma. Scale bars, *A–H*: 10 µm.

**Figure 4:**
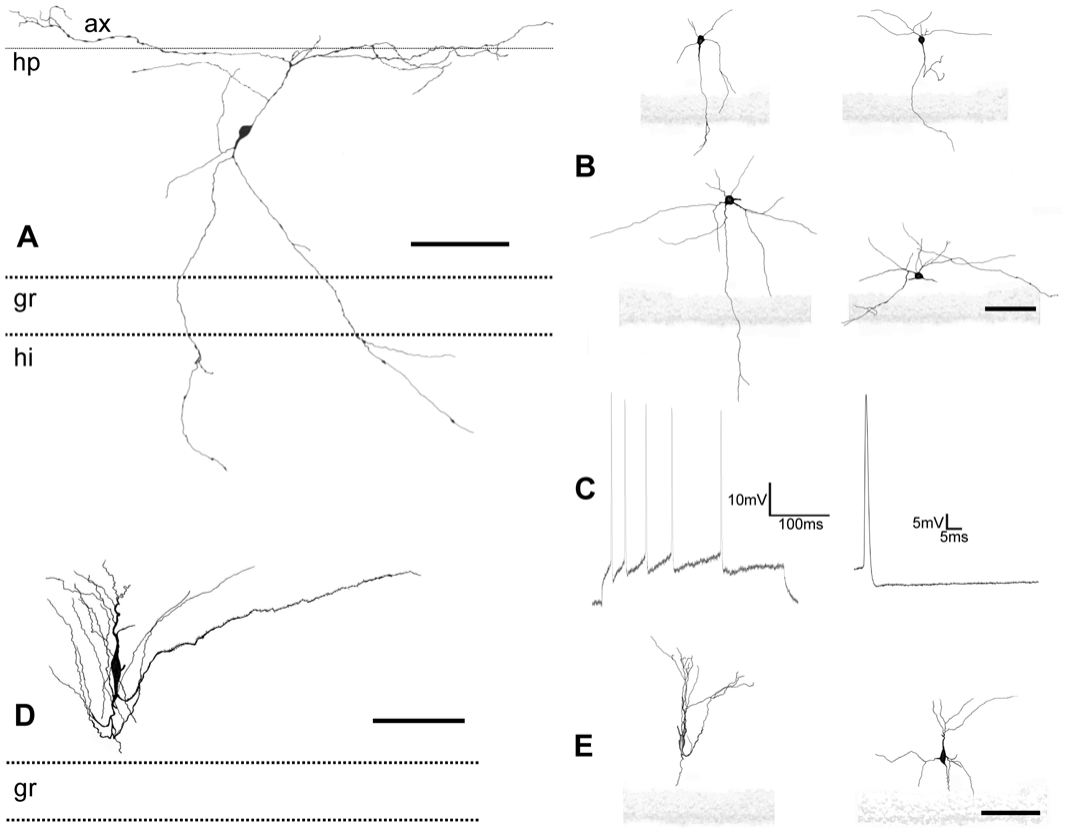
Camera lucida drawings and electrophysiological recording of globoid and vertical neurons. *A,* Globoid neuron with axon (**ax**) in the hippocampal fissure (**hp**) and dendrites crossing the granular layer (**gr**) reaching the hilus (**hi**). *B,* drawings of different globoid neurons. *C,* electrophysiological recording of action potentials in a globoid neuron elicited by a stimulus of 300 ms. *D, E,* drawings of vertical neurons. Scale bars, *A–E:* 25 µm.

## Results

Nissl stainings allowed us to easily distinguish and count cell bodies in the molecular layer (Figure S1). The number of neuronal somata in the molecular layer of rabbits varied among individuals; 14447±4667 neurons on average per hemisphere. It increased from newborn PO postnatal animals (5302±87 neurons) until acquiring the final size at about one week afterwards (15211±4346 neurons in P6 animals). No significant difference was observed in the other age groups (young, adult, old) (Fig. 1G), thus indicating that the rabbit molecular layer contains a steady/permanent neuronal population. Nevertheless glial cells underwent changes during postnatal development, i.e., abrupt loss in P12–P15 animals followed by significant increases in P15–P30 animals (Fig. 1H–K). The phenomena that may be connected with intensive synaptic rearrangement in the areas following two critical postnatal events are: eye openings about P10–P11 and weaning about P17.

Eight different neuronal archetypes were clearly distinguished in both the Golgi preparations and the NADPH histochemistry of perinatal, young, adult and old animals. According to their morphology, they were named globoid, vertical, small horizontal, large horizontal, inverted pyramidal, polymorphic neurons, sarmentous and ectopic granular. Immunostainings and intracellular injections of biocytin in slightly fixed brain slices were used to assess this morphological classification, and also allowed the electron microscopic study to identify gabaergic neurons. In addition, morphological and firing pattern correlation was performed by filling neurons after electrophysiological recordings.

**Figure 5:**
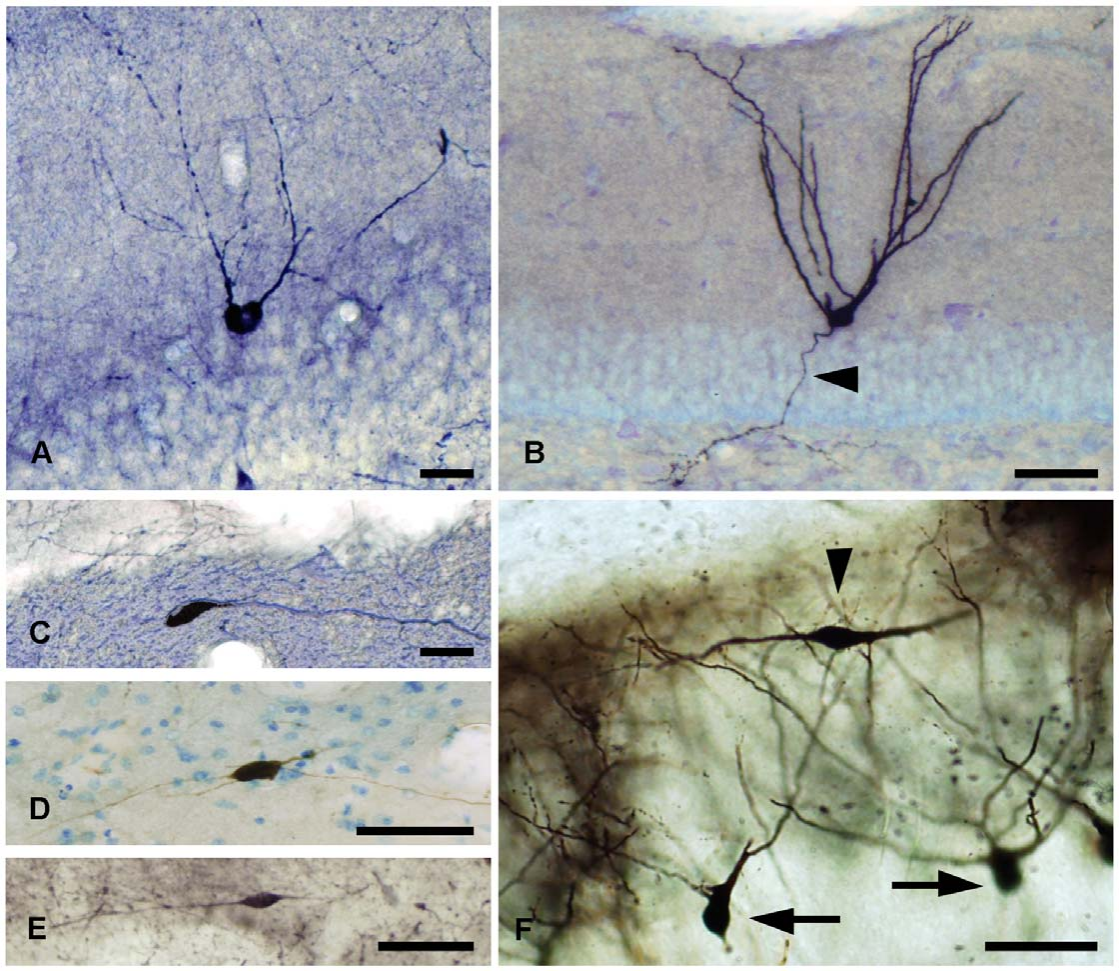
Stainings of ectopic granular and small horizontal neurons. *A,* NADPH diaphorase histochemical staining of an ectopic granular neuron. *B,* ABC-diaminobenzidine-nickel staining of biocytin injected into a granular ectopic neuron with an axon (arrow head) crossing the granular layer. *C,* NADPH diaphorase histochemical staining of a small horizontal neuron. *D,* idem, parvalbumin immunostaining. *E,* idem, calretinin immunostaining. *F,* Golgi impregnated small horizontal neuron (arrow head) and two granule cells (arrows). Scale bars, *A–F*: 25 µm.

**Figure 6:**
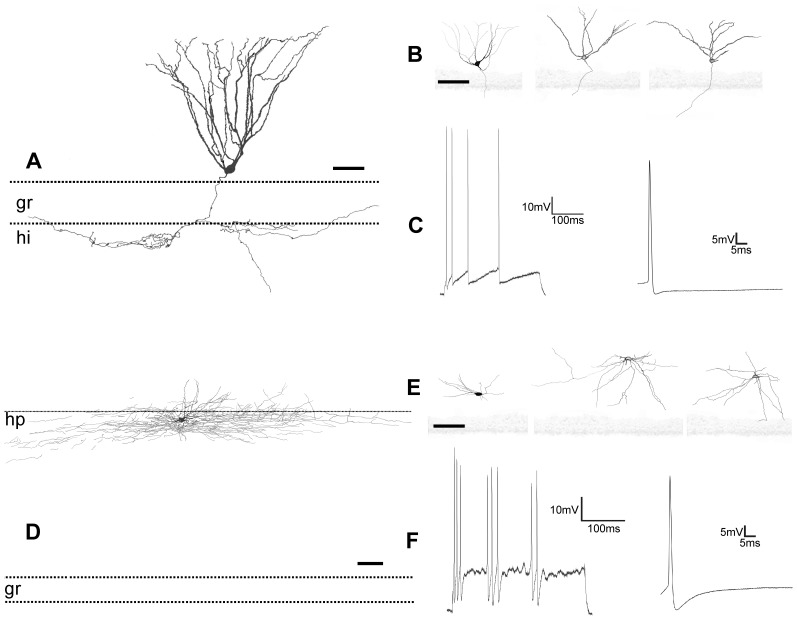
Camera lucida drawings and electrophysiological recordings of granular ectopic and small horizontal neurons. *A, B,* drawings of ectopic granular neurons in A, the axon crossing the granular layer, bifurcates and arborization in the hilus (**hi**). *C,* electrophysiological record from an ectopic granular neuron. *D, E,* drawings of the small horizontal neurons. Observed in *D*, the profuse axonal arborization close to the hippocampal fissure (**hp**). *F,* electrophysiological recording of a small horizontal neuron. Scale bars, *A–F:* 25 µm.

### Globoid Neurons

These neurons presented spherical calbindine (Fig. 2A) and GABA (Fig. 3A) immnunoreactive somata and very thin spine-free and not excessively branched dendrites (Fig. 4A–B). One or two of them ran through the granular cell layer to finish in the hilus (Fig. 4A–B). Their axonal arbours were located in parallel to the hippocampal fissure. Globoid cells were very frequently observed in the NADPH diaphorase stainings, displaying a relative frequency of close to 50% of all NADPH positive. In the electron microscope (Fig. 3A.1) they displayed nuclei with abundant eucromatin, slightly displaced nucleoli and a thin cytoplasmic stripe with free ribosomes, some RER cisterns, dictiosomes, etc. Their response to depolarizing current injection pulses was a train of spikes with similar values for amplitude and duration, with frequency accommodation, very similar to displaced granule cells (Fig. 4C).

**Figure 7:**
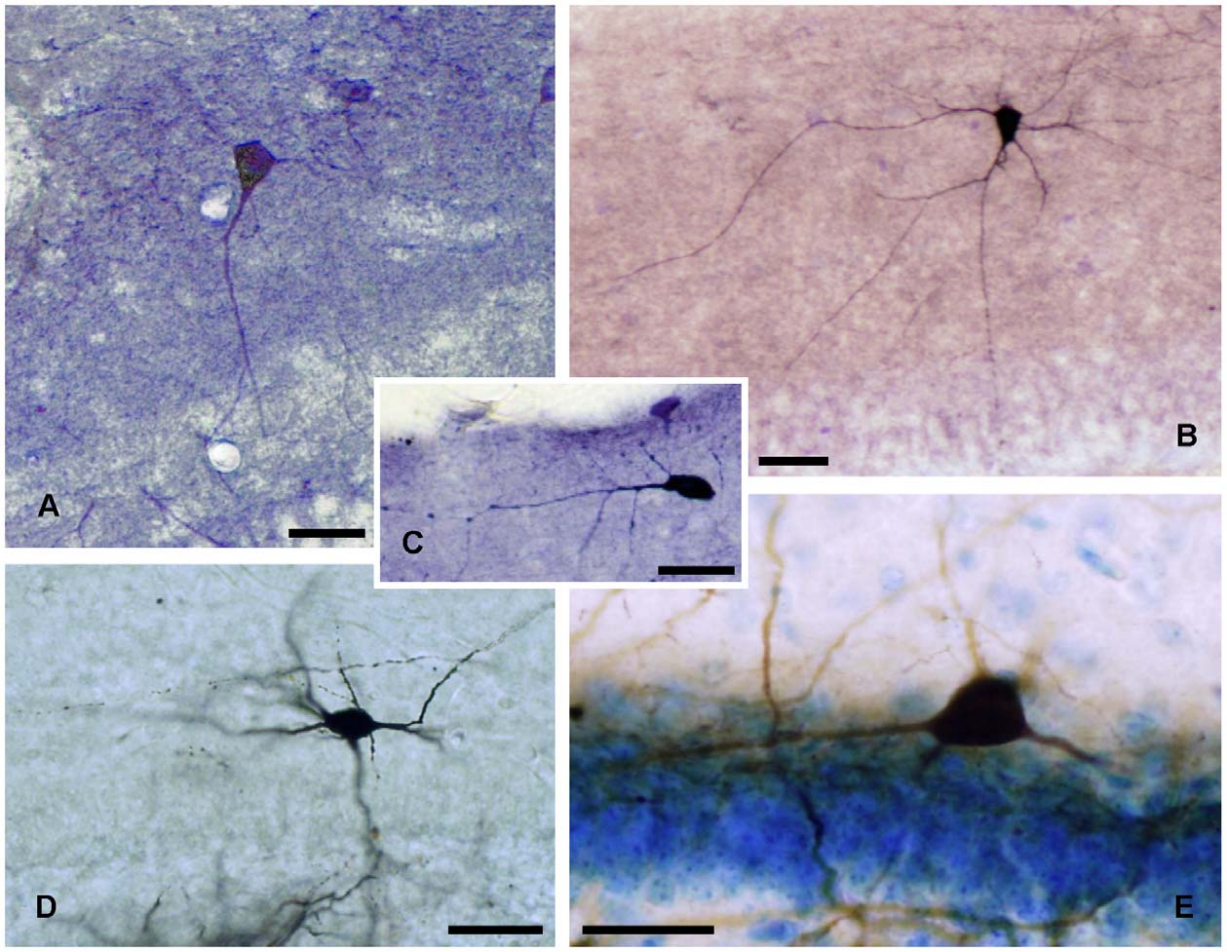
Stainings of inverted pyramidal and large horizontal neurons. *A,* NADPH diaphorase histochemical staining of an inverted pyramidal neuron. *B,* ABC-DAB-nickel staining of biocytin injected into an inverted pyramidal neuron. *C,* NADPH diaphorase histochemical staining of a large horizontal neuron in the inferior blade of the dentate gyrus. *D,* Golgi impregnation of a large horizontal neuron. *E,* parvalbumin immunostaining of a large horizontal neuron in the superior blade of the dentate gyrus; note the counterstained granular layer. Scale bars, *A–E*, 25 µm.

**Figure 8:**
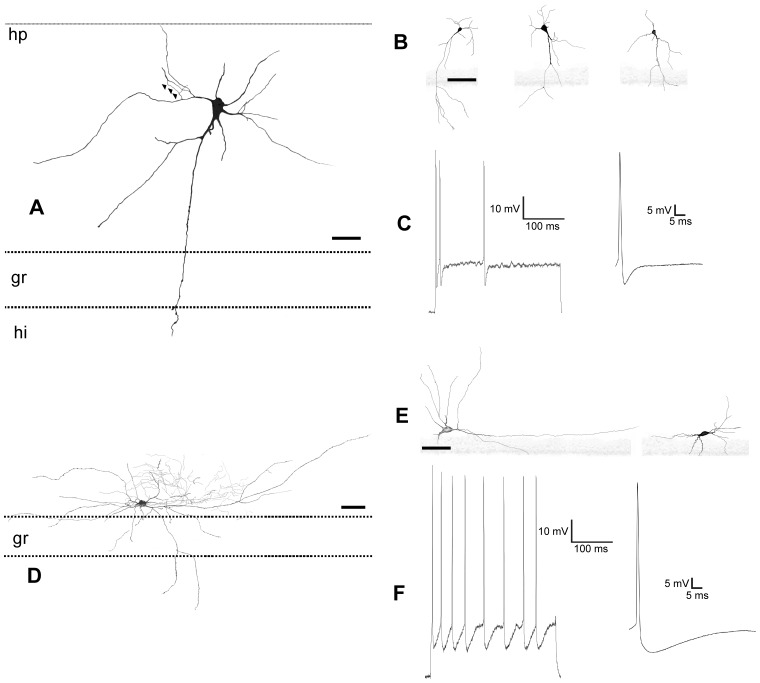
Camera lucida drawings and electrophysiological recordings of inverted pyramidal and large horizontal neurons. *A, B,* Drawings of inverted pyramidal neurons. The neuron in *A* is that shown in Fig. 6B; note the place of the axonal hillock marked by triangles. *C,* electrophysiological recording of an inverted pyramidal neuron. *D, E,* drawings of large horizontal neurons. *F,* electrophysiological recording of a large horizontal neuron. Scale bars, *A–E:* 25 µm.

**Figure 9:**
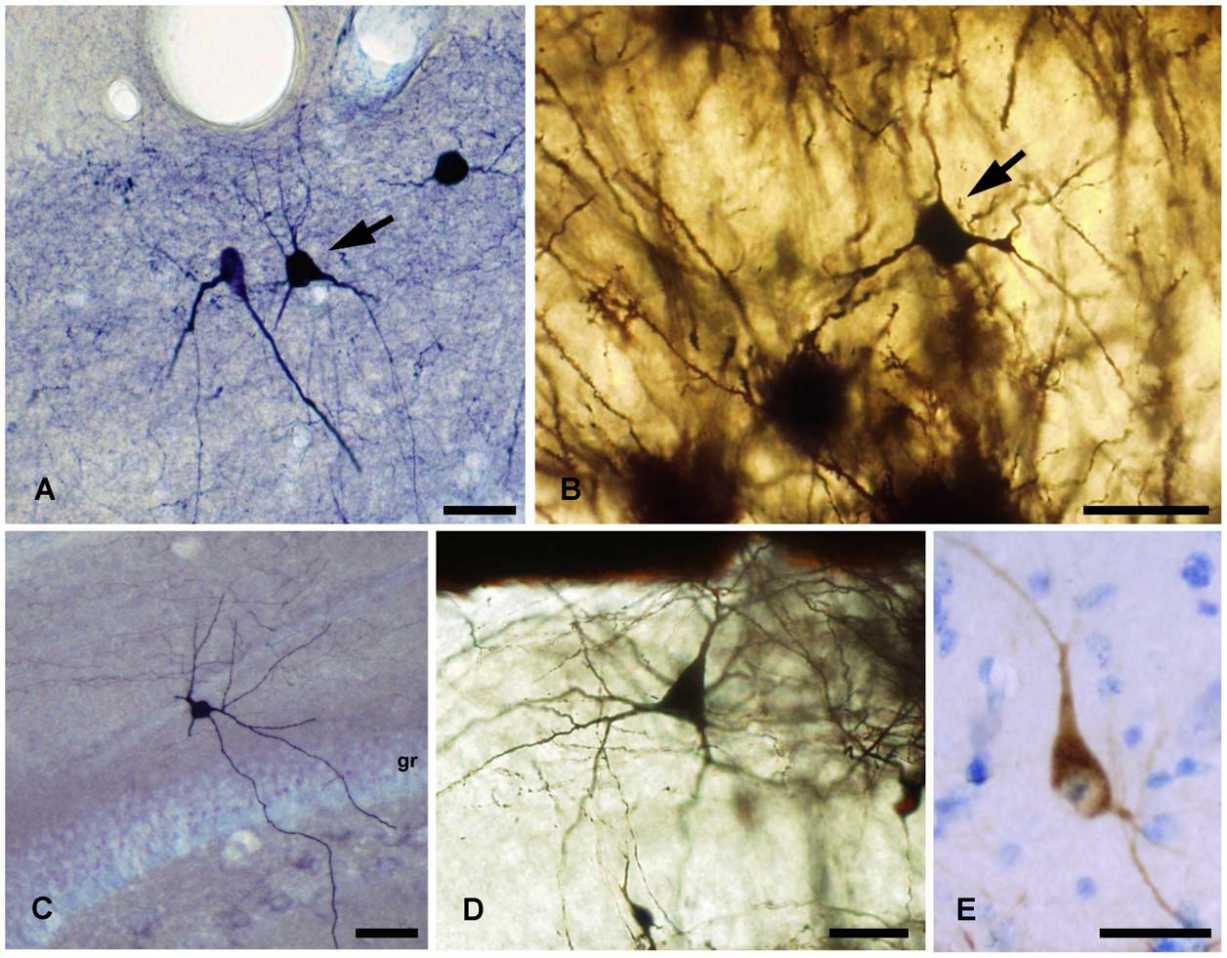
Stainings of sarmentous and polymorphic neurons. *A,* NADPH diaphorase histochemical staining of a sarmentous neuron (arrow) (inverted pyramidal on the left and globoid on the right). *B,* Golgi impregnation of a sarmentous neuron (arrow). *C,* ABC-DAB-nickel staining of biocytin injected into a polymorphic neuron with two dendrites crossing the granular layer (**gr**). *D,* Golgi impregnation of a polymorphic neuron. *E,* parvalbumin immunostaining of a polymorphic neuron, toluidine blue counterstaining. Scale bars, *A–E:* 25 µm.

### Vertical Neurons

These neurons were GABA (Fig. 3C) and calretinin immunoreactive (Fig. 2D), had a conspicuous vertically oriented fusiform soma and two dendritic tufts with the same orientation (Fig. 4D–E). The upper tuft of dendrites ran toward the hippocampal fissure, and the lower tuft ran toward the granular cell layer. Some of the lower tuft dendrites turned to the hippocampal fissure before touching the granular cell layer. Occasionally, scarce dendritic spines appeared in the main dendrite (Fig. 4D–E). The relative frequency of the vertical neurons in the NADPH diaphorase preparations was quite low, close to 1.2%. No bioelectrical recordings were obtained from this type of neurons.

**Figure 10:**
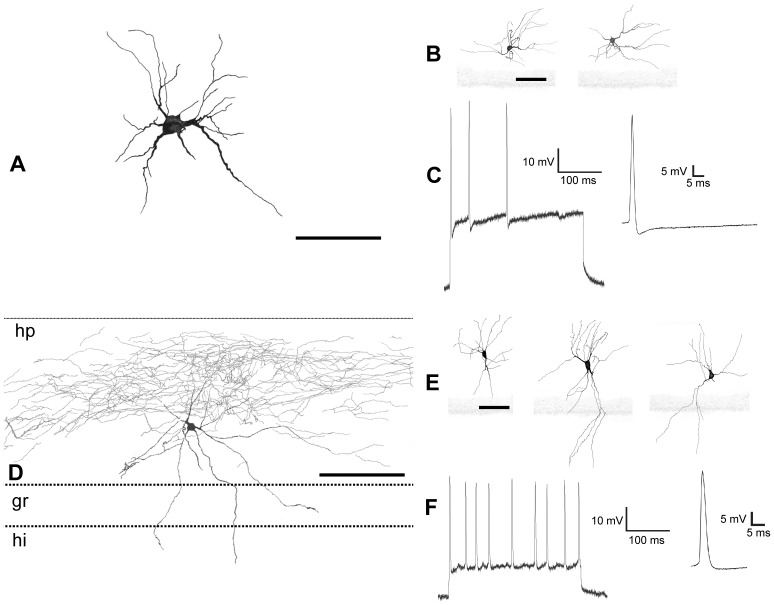
Camera lucida drawings and electrophysiological recordings of sarmetous and polymorphic neurons. *A, B,* Drawings of sarmentous neurons. *C,* electrophysiological recording of a sarmentous neuron. *D, E,* drawings of polymorphic neurons; note the dense axonal arborization in *D* distributed by the outer and middle molecular layers. *F,* elecrophysiological record of a polymorphic neuron. Scale bars, *A–E:* 25 µm.

**Figure 11:**
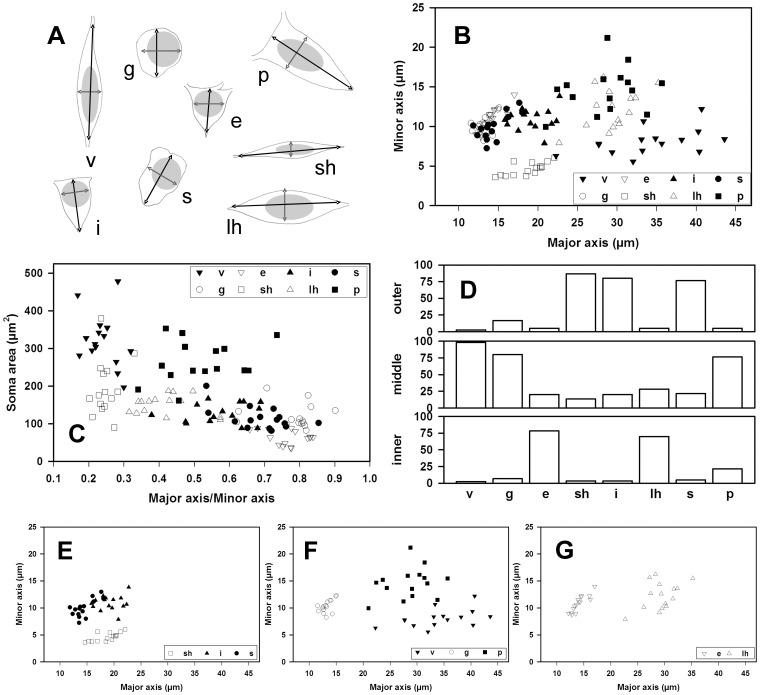
Morphometry and distribution of the neuronal somata in the molecular layer of the dentate gyrus. *A,* Profiles of neuronal somata from different neurons taken as prototypes; the length of the two principal axes and the perimeter length were the main parameters analyzed, (v- vertical neuron, g- globular neuron, e- ectopic granular neuron, sh- small horizontal neuron, lh- large horizontal neuron, s- sarmentous neuron, P- polymorphic neuron). *B,* Frequency of location of neuronal types in the inner, middle and outer molecular layer strata (from a pool of 120 neurons from a 30-day-old rabbit; 15 neurons/type). *C,* Plots of the minor axis value against the major axis value; observe how specific neurons segregate in distinct populations. *D,* Idem, plots of the surface value of the profile (soma area) (ordinate) against the axes ratio (minor/major) and some types also segregate. *E,* Plots of the minor axis values (abscise) in front of the major axis values (ordinate) of the neurons which are more frequent in the outer molecular: sarmentous, inverted pyramidals and small horizontals. *F,* Idem, for the more frequent neurons in the middle molecular: verticals, globoid and polymorphic. *G,* Idem, for those more frequently located in the inner molecular: ectopic granular and large horizontal. Scale bars, *A–G*: 25 µm.

**Figure 12:**
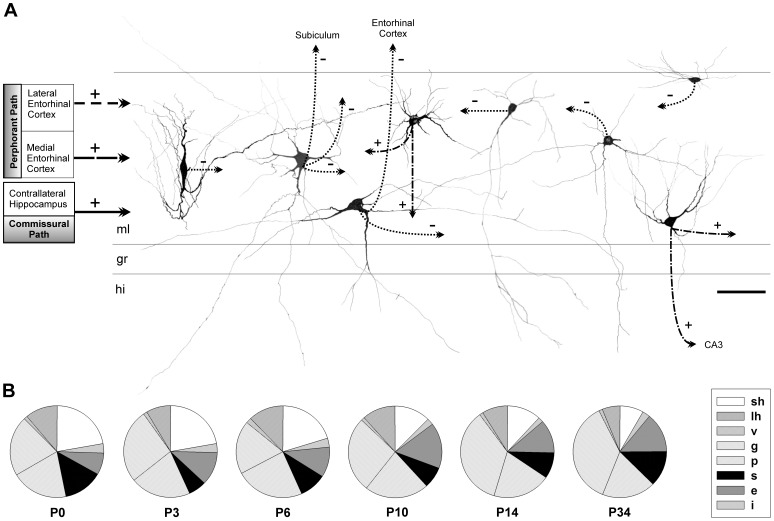
Diagram of distribution and putative role of the eight identified archetypes in the hippocampal circuitry, and their temporal evolution in the early postnatal period. *A,* Drawing composed of individual camera lucida reconstructions located according to the archetype preferred position in the layer. From left to right: vertical, polymorphic, large horizontal, sarmentous, inverted pyramidal, globoid, ectopic granular and small horizontal neuron. In addition, this drawing shows the main afferent and efferent projections of each neuronal which suggest reveal their putative roles in the hippocampal circuitry. The inhibitory or excitatory nature of each neuronal archetype is represented by (−) and (+) symbols respectively. Scale bars, 25 µm. *B,* Relative frequency distributions at P0, P3, P6, P10, P14 and P34 which show a moderate increase of globoid neurons and a simultaneous decrease of small horizontals.

### Ectopic Granular Neurons

They presented occasional calbindin immunoreactivity (not shown), but were GABA negative (Fig. 3D). They appeared in the NADPH preparations (Fig. 5A) with an average frequency of 7%. However, their frequency apparently increased with age (i.e., in Nissl stainings, from 10% P10 rabbits to 13.7% in P34 rabbits). The response to depolarizing pulses was a train of spikes with a marked spike frequency adaptation, and large and constant amplitudes (Fig. 6C).

**Figure 13:**
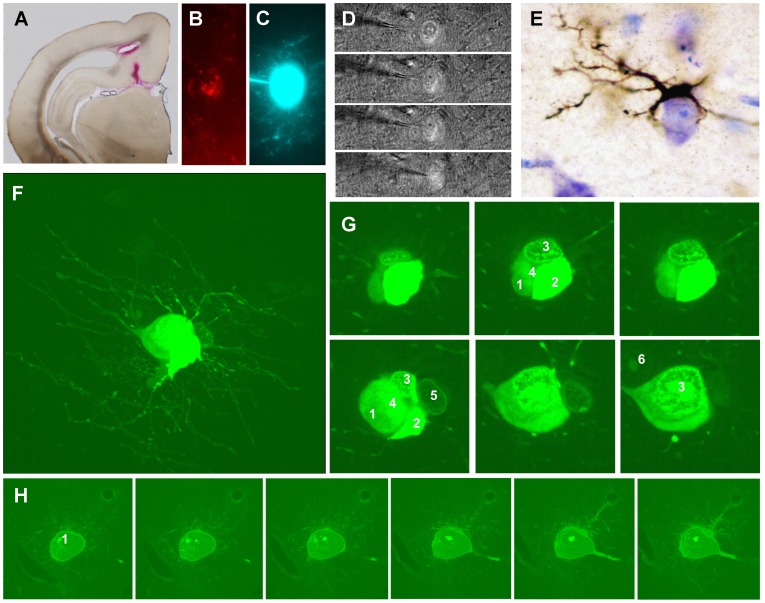
Retrogradely labeled neurons in the molecular layer of the rabbit gyrus dentatus. *A,* Injection place of DiL; note the red track of the microsyringe in the subiculum. *B,* Red fluorescent labeling in a cell soma located in the molecular layer (40×). *C,* Impalement of cell somata and the injection of Lucifer Yellow; note the bright signal from the soma and from some thin projections. *D,* Different depth focus plane of the impaled soma and surroundings (double illumination with transmitted light plus DiL fluorescence); note the presence of several cell somata closely attached to the labeled soma. *E,* Microglia cell surrounding a toluidine blue stained neuronal soma in the molecular layer of an 8-year-old rabbit; (Timm-Danscher staining for ionic zinc). *F,* Confocal microscopic image of a retrogradelly labeled soma and associated glial cells; observe the very thin cell processes from an oligodendrocyte attached to neuronal somata whose processes appear less intensively labeled. *G,* Confocal planes pictures of the cell shown in *F* (3); observe the close attachment of up to 6 cell somata with different histological appearances, one of which is clearly identified as oligodendroglia (2). *H*, Confocal plane pictures from a retrogradely labeled neuronal soma in the molecular layer, also displaying attached glial cells; here one of the glial cells (3) is an astrocyte with a vascular end foot process. Scale bars, *A:* 100 µm; *B–H:* 10 µm.

### Small Horizontal Neurons

These were GABA (Fig. 3H) and calretinin (Fig. 5E) immunoreactive neurons with fusiform somata located parallel and very close to the hippocampal fissure which occasionally displayed parvalbumin (Fig. 5D) immunoreactivity. This type of non spiny neuron (Fig. 5F) usually had a principal thin dendrite arising from the soma which could bifurcate. The axonal arbor was distributed under the hippocampal fissure (Fig. 6D–E). In the first month of postnatal life, these neurons decreased numerically in NADPH preparations from 20% in postnatals to 10% in adults, but they did not disappear (Fig. 5C). They responded very differently to current injections with a series of two to three phasic burst of spikes with each burst separated by oscillation periods (Fig. 6F).

### Inverted Pyramidal Neurons

They were GABA immunoreactive neurons (Fig. 3E) with pyramidal somata whose presumed apical pole was directed toward the granular layer (Fig. 7A–B). From the upper parts of the soma, a couple of thin dendritic tufts arose and directed toward the hippocampal fissure. From the lower vertex, one or two main dendrites arose and ran to the interior crossing of the granular layer (Fig. 7A–B). They were non spiny and their axon ramified very early and gave rise to a profuse axonal arborization localized in the middle and outer strata of the layer (Fig. 8A–B). They were frequently noted in the NADPH diaphorase preparations (close to 20%) (Fig. 7A), but less frequently in the Nissl stainings (3–4%). Their response to intracellular electrical stimulation consisted in a burst of two or three action potentials followed by a single spike (Fig. 8C). The first action potentials presented larger amplitudes than posterior ones and it was possible to observe the membrane potential oscillations during the current pulse.

### Large Horizontal Neurons

These neurons were GABA (Fig. 3G) and parvalbumin (Fig. 7E) immunoreactive with large fusiform somata located parallel and very close to the granular cell layer. The principal dendrites emerged from the apical poles and also ran in parallel to the granular cell layer (Fig. 7D). Normally one or two dendrites crossed the granular cell layer and entered the hilus (Fig. 8D–E). The axonal arbor was located in the inner stratum of the molecular layer. The relative frequency in the diaphorase preparations was lower than 5% (Fig. 7C). The response to current injection (Fig. 8F) consisted in a train of action potentials with a tonic firing pattern. Spike amplitude remained constant and no accommodation was observed.

### Sarmentous Neurons

These neurons had an irregularly shaped soma with some thick dendritic trunks from which long thin dendrites arose (Fig. 9A–B). Dendrites did not seem to prefer a specific direction and occasionally a few dendritic spines appeared near the soma. Axons emerged from the soma, ramified by the outer and intermediate strata, and gave rise to collaterals that crossed the hippocampal fissure (Fig. 10A–B). These neurons were negative for GABA (Fig. 3B) immunostaining and optionally expressed calbindine (not shown) immunoreactivity. They were frequent in the diaphorase preparations (almost 10%) (Fig. 9A). Their response to depolarizing pulses was a train of action potentials with marked spike frequency adaptation (Fig. 10C).

### Polymorphic Neurons

These neurons presented large GABA (Fig. 3F) and parvalbumin (Fig. 9E) immunoreactive somata from which thick dendrites arose and went toward any direction. Indeed, one or two of these dendrites ran across the granular cell layer. This was either a non spiny neuron with axons arising directly from the soma or a principal dendrite which occupied the outer two thirds of the molecular layer (Fig. 9C–D, 10D–E); axonal branches crossed the hippocampal fissure. Their relative frequency in the diaphorase preparations was close to 5% (not shown). When current pulses were intracellularly injected, the firing pattern consisted in a train with no evident adaptation. Spikes presented the smallest amplitude in the group (Fig. 10F).

### Morphometric Study and Layer Distribution

The study of the biometrical sizes and shapes of the neuronal somata in Nissl staining assessed the morphological classification done with NADPH-diaphorase, Golgi impregnations and intracellular injections. The neurons with a higher soma surface/perimeter were the polymorphic neurons, while the smaller soma surface corresponded to small horizontal neurons, but not the minor perimeter, which was shown by globoid neurons due to these cells being round and spherical (Fig. 11A–C). Described neuronal types could be distinguished from NADPH and Nissl staining by analyzing the major (D) and the minor (d) diameters and soma surface (Fig. 11A–C).

Finally, segregation was completed by considering the position of the soma in the inner, middle or outer sublayer (Fig. 11D). This allowed the identification of the neuronal type of each neuronal somata in the Nissl stainings (Fig. 11E–G) to, thus, deduce their relative frequencies which were closer to the actual values than those observed in the NADPH preparations.

Taking together all previous data, distribution and putative role in the hippocampal circuitry of the eight molecular layer neuronal archetypes may be proposed according to their preferred position in the strata (Fig. 12A).

The analysis of soma size plus shape archetypes in Nissl-stained serial sections from animals of different ages allowed deducing the evolution of the neuronal types in the dentate molecular layer throughout postnatal development (Fig. 12B). A marked decrease was observed in the proportion of the small horizontal neurons until the third postnatal week, which did not disappear altogether. The changes noted with the large horizontal neurons were not so marked, although the population lowered slightly. Populations of vertical and pyramidal inverted cells remained constant. Globoid neurons increased, starting from P14. The polymorphic neurons population remained constant, while the sarmentous neurons population varied among ages without following a clear pattern of change. Finally, the ectopic granular neurons increased slightly with age.

### Retrograde Labeling

In order to assess whether molecular layer interneurons send axonal branches outside the dentate gyrus we injected markers for retrograde transport in two cortical areas: the entorhinal cortex and the subiculum. We found negative results when injecting tracers in the entorhinal cortex. Nevertheless local injections of DiL in the subiculum resulted in the retrograde labeling of some neuronal somata in the molecular layer (Fig. 13A–C). The intracellular injection of these somata revealed that they were large polymorphic and sarmentous neurons with attached glial satellite cells of varying appearances (Fig. 13D). Frequently, glial cells were filled with the tracer as the micropipette passed through them to impale the retrogradely labeled neuron (Fig. 13F–G). While some of these attached cells were microglia, others were oligodendroglia and even astroglia (Fig. 13H). A confocal study of these cell groups suggests the possibility of all the glial types coexisting around all the retrogradely labeled somata. The somata of the molecular layer of very old animals were surrounded by microglia cells, which was evidenced with zinc histochemical autometallography (Fig. 13E).

## Discussion

In the first descriptions on the neuronal morphology of the rabbit molecular layer [2], Ramón y Cajal described two groups of cells *“triangular cells or displaced granular”* and *“short axon cells”* divided into two other groups, superficial and deep cells, with several somatic morphologies. If we look at the author’s drawings, we can observe up to nine different neuronal morphologies. The eight neuronal archetypes described herein are evident. Vertical, horizontal, polymorphic, inverted pyramidal and sarmentous archetypes are so conspicuous that they can be recognized at first sight in Golgi and diaphorase preparations or with any Golgi-like immunostaining, and even in Nissl stainings after biometrical appreciations.

The only problem in assigning a correct archetype for a given neuronal soma occurs when Nissl or Nissl-like stained somata display shapes between globoid and displaced granule archetypes as they apparently share similar dendritic tree patterns and soma sizes. Even some immature oligodendrocytes may be also confused. But the observation of their dendrites, when possible, i.e., with variable thickness and aboundant dendritic spines versus long, slender and almost constant thickness, and differential GABA immunoreactivity resolved the problem and clearly segregated the identity of both types.

The overhelming majority of neurons in the molecular layer may be considered as “interneurons”, as they display GABA immunoreactivity and characteristic dendritic shape (non-spiny dendrites).

Besides from the granular ectopic neurons, the only GABA negative neurons of the rabbit molecular layer are those here called “sarmentous” which may be regarded as the true “aborigine” neurons of the layer and the rest of neurons being immigrants come after relatively long journeys [21], [22].

Some of these morphological archetypes may be recognized in other vertebrate species, for instance, the medial cerebral cortex of lizards [23] from which the name “sarmentous” was taken; even similarities between lizard and rabbit neurons may be extended to other types, i.e., lizard stellate, lizard deep stellate neurons and lizard “couchant” resemble the rabbit globoid, rabbit multipolars, and rabbit large-horizontals, respectively. The criteria to distinguish them have been mainly of a morphologic nature. Nevertheless, the additional electrophysiological study has evidenced the diversity of the molecular layer in the neuronal population. The six stereotyped responses after a depolarizing stimulus are apparently characteristic of non principal neurons (interneurons), a characteristic that fits the GABA immunoreactivity of the vast majority of them, with the exception of the sarmentous and ectopic granule cells.

Inverted pyramidal neurons have been described in the inferior layers of the rabbit visual cortex [24] whereas ectopic granule cells have been described in rodents [10] and were drawn in the earliest Ramón y Cajal studies [2].

Small horizontal neurons seem to be Cajal-Retzius neurons, which are very abundant in the early postnatal periods but then dramatically decrease in the first postnatal weeks. The cause of this phenomenon has been pointed out in rodents: the incoming glutamatergic perforant axons from the entorhinal cortex cause excitotoxicity in their allurement neurons: the Cajal-Retzius cells expressing glutamatergic receptors [12]. Nevertheless, it is important to note that, in rabbits, some small horizontal neurons still survive and remain in the adult brain.

Another developmental variation is that which concerns the compensatory increase of globoid and granular ectopic neurons with postnatal age. The slight increase of ectopic granule cells noted may be the result of migratory errors after the neurogenetical activity of the subjacent subgranular layer [25]. The increase of globoid neurons appears to be enigmatic; together with inverted pyramidals, they appear near blood vessels and display NADPH diaphorase and NOS activity [26], probably in relation to blood flow control.

In the rabbit, and probably in the rest of mammals, most interneurons of the molecular layer occupy their places during the very early stages of embryonic development [4]–[7]. Since they are GABA immunoreactive interneurons, they are likely to be born in the median eminence during early development and then distribute to the molecular layer through large tangential migrations [22], [27]–[29]; the small calretinin immunoreactive Cajal-Retzius horizontal neurons may represent the final wave of this tangential migration. Even the presence of vertical neurons may also be a result of the unfinished final step of long tangential trajectories from the hippocampal subventricular zone [22] which end in short radial courses before reaching their final position [21], [30].

Nevertheless, we hypothesize a similar histogenetic process for globoid and granular ectopic neurons: they are generated in the subjacent subgranular layer during late embryonic development and are then radially displaced to the molecular layer. It is also possible that *semilular granule cells* (SGCs) in the IML represent “elderly” granule cells (GCs) [31].

DiL retrogradely labeled somata are identified as polymorphic neurons and can be compared to the rodent MOPP (“molecular outer perforant path”) neurons [32] with the somata in the inner molecular layer and axonal arborizations in the middle/outer molecular layer and beyond the hippocampal fissure. There is no clear explanation for the associations of glia cells with DiL retrogradely labeled somata. Glial cells associate with neuronal somata would be stained as a result of some signal or damage induced by retrograde labeling. Microglia cell staining with zinc histochemical autometallographic techniques has been previously described in rats [33]. In this study, the zinc staining of perisomatic microglia only occurred in very old rabbits, which may be indicative of a hypothetical age-related degenerative process [34].

### Position in the Hippocampal Circuitry

Most neuronal types in the molecular layer are non spiny and GABA immunoreactive while GABA negative neurons are sarmentous and granular ectopic cells, as deduced after electron microscopic studies. Since the main input to them is derived from the perforant and commisural axons, these interneurons may exert an immediate feed-forward inhibitory effect on the molecular layer, this being the main gate entrance for the hippocampus. Long feed-forward inhibition onto the subiculum via the long axonal collaterals of polymorphic neurons may also be presumed. Feed-back inhibitory projections to the entorhinal cortex may be another possibility in rabbits, as shown in rats [14], but we were unable to provide evidence for this.

In short, the outer molecular layer of the rabbit fascia dentata has a scarce but well defined and heterogeneous neuronal population, mainly formed by GABA immunoreactive somata, which remains until the animal is old, but with slight postnatal changes in the numbers and frequency of the different types.

## Materials and Methods

### Animals

All the animals were housed under a 12 h light/dark cycle with food and water available *ad libitum*. All animal procedures were reviewed and approved by the Ethical Committee for Use of Laboratory Animals of the University of Valencia, and followed the European Communities Council (86/609/EEC) guidelines. All efforts were made to minimise animal suffering and to reduce the number of animals used. Fifty two postnatal (P0, P3, P6, P8, P10, P12, P14, P16, P18, P20, P24, P36 and P60 day-old), five adult (aged 1 and 2 years) and five old (aged 6 to 8 years) New Zealand white HY/CR female rabbits (*Orictolagus cuniculus*) from an authorized supplier (Iffa-Credo, Lyon, France) were used in the histological study. Fifteen additional P20 animals were used for electrophysiology and intracellular injections into live slices. Animals were obtained from an authorized supplier (Iffa-Credo, Lyon, France).

### Additional Material

A laboratory collection of over hundred Golgi-impregnated rabbit brains following the *Colonier* variant procedure [23] of all the developmental ages (from P0 to 6.5 years), and Timm-stained transversal serial sections, were used to successfully observe impregnated dentate gyrus molecular layer neurons.

### General Procedure for Histology

Animals were anesthetized (pentothal sodium; 60 mg/100 g b.w.) and perfused intracardially by using a Cole-Palmer Masterflex pump at different speed/pressure (6 ml/min for perinatals and 25 ml/min for young and old specimens). Buffered saline solution (PBS), 0.01 M sodium phosphate buffer in saline solution 0.9% sodium chloride (optionally, heparinized saline solution (25 IU/ml in 0.9% Ringer solution) was used to clear blood vessels. The fixative used was a fresh mixture of two solutions: 4% paraformaldehyde (PFA) and 4% glutaraldehyde (GA) in 0.1 M phosphate buffer pH 7.2–7.4, for 20 min., usually a 1∶1 mixture (optionally only PFA for immunohistochemistry or different PFA:GA proportions of about 1∶3 to 3∶1 for intracellular injections into animals of different ages, see below).

After dissection and brain extraction, brain hemispheres were split and processed separately. One hemisphere for histological study was postfixed overnight in the same fixative, cryoprotected in sucrose 30%, and transversally sectioned (50 µm thick) in parallel series with a freezing microtome. For intracellular injections in fixed slices, the hippocampus of the other hemisphere was immediately dissected and transversely sectioned using a vibratome (slices of 100, 200 or 300 µm).

### Nissl Staining and NADPH-Diaphorase Histochemistry

For Nissl staining, 50 µm-thick sections mounted on gelatine-coated slides were incubated in toluidine blue pH 4.1 for 1 min, dehydrated through a battery of alcohols with increasing graduation, and coverslipped in Eukitt.

NADPH-Diaphorase histochemistry was carried out by incubating (for 60–90 min at 37°C) vibratome tissue sections in Tris HCl buffer 0.1 M, pH 8.0 with nitrotetrazolium blue (0.065% w/v), β-NADPH tetrasodium salt (0.1% w/v) (Sigma) and Triton X-100 (0.4% v/v). The reaction was stopped with several rinses in Tris HCl buffer 0.1 M, pH 8.0. Sections were then washed, mounted on gelatine-coated slides, dehydrated, coverslipped in Eukitt and observed under a light microscope.

### Stereological and Biometrical Procedures

Counts of neuronal and glial cell somata appearing in the molecular layer of the 50 micron-thick Nissl counterstained sections were done by using a 40X objective and following a simplified version of the stereological *Optical Fractionator* method [35]. In short, counts of the cell somata appearing in the sampling area. Cells appearing in the upper focal plane were omitted to prevent overcountings. Counts were systematically done at sampling points during sequential movements of 200×200 microns in the X-Y plane along the 50 micron-thick section. The estimation of the total number of cells in the layer was done with the following formula:
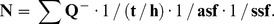
Where t = h was the countings done in the complete thickness of the 50 micron-thick section, asf (area sampling fraction), ssf (sections sampling fraction). The variance values of four repeated estimations in one animal series with this systematic sampling (sampling points at 200X200 microns, in one section every ten) were by far inferior to the half values of the variance between estimations for animals of the same developmental stage. Thus the sterological procedure was considered valid. The number of sampling points in every hippocampus varied from 23 in P0 postnatal animal to 316 in adult rabbits; the number of neuron/glial profiles per sampling area varied from 1.1/7.4 in postnatal animals to 0.6/4.1 in adults. Means were determined for each experimental group.

In order to find the best systematic sampling procedure we repeated estimations in a same animal series by changing sampling points (jump steps from 200X200 microns to 400X400 microns) and by increasing the section sampling fraction (from one section every 10 to every 20 sections in the series). Obviously the less variance values were obtained with the most strict procedure (jumps of 200X200 microns, one section every ten).

Biometrical measures of cell somata were done in Nissl stained preparations from both postnatal and adult specimens, at least fifty cell pictures for each neuronal type, obtained with an Olympus Dp-70 camera. The different parameters evaluated were measured with the appropriate software (Image Tool, v1.0). The final values for somata larger and smaller axes, perimeter and area were expressed as mean ± SD.

### Immunostainings for Calbindin, Calretinin and Parvalbumin

Subsets of free floating sections, either coming from the freezing microtome or from the vibratome, were processed for immunodetection. In short, after washing and blocking treatments, sections were overnight incubated in the correspondent primary antibody: calbindin 1∶1000 diluted monoclonal mouse anticalbindin antibody (Sigma, St Louis, MO); calretinin in 1∶7500 diluted goat anticalretinin antibody (Swant, Bellinzona, Switzerland); and parvalbumin in 1∶1000 diluted monoclonal mouse antiparvalbumin antibody (Sigma, St Louis, MO). Antigen-antibody complexes were visualized using either 1∶200 diluted biotinylated rabbit-anti-goat IgG for primary goat antibody or goat anti-mouse for the primary mouse antibody (Sternberger Monoclonals Incorporated, Lutherville, Maryland), and the Vectastain ABC immunoperoxidase kit (Vector Laboratories, Burlingame, CA). All the immunoreagents were diluted in phosphate-buffered saline containing 5% normal goat serum, 0.2% gelatine, and 0.1% Triton X-100. The immunoperoxidase reaction was developed using 3,3′-diaminobenzidine as a chromogen. Then, sections were washed, mounted on gelatine-coated slides and coverslipped in Eukitt. Subsets of sections belonging to different aged specimens were processed simultaneously to avoid undesired methodological differences in the immunostaining. Some control sections of every animal were processed in the same way, but primary antibodies were omitted. No immunostaining was observed under these conditions.

### Intracellular Injections in Slightly Fixed Slices

Animal brains were obtained after intracardial perfusion with variable GA + PFA mixtures for different animal ages (from GA 2.5% + PFA 0.8% for P0 to GA 1% + PFA 0.3% for P25). The brain was removed and the hippocampus dissected as previously described. Then transversal 200–300 µm-thick slices were obtained with a vibratome (Leica VT1000S) and stored in 4% PFA in PB 0.1 M pH 7.2 at 4°C until successful injections were completed (12 h after perfusion at the latest).

An Olympus BX50WI microscope was used, equipped with long distance 10X and 40X water immersion objectives, a digital TV camera (Hamamatsu) and a TV screen to monitor how micropipettes (150–200 MΩ resistance, filled with the tracing solution Lucifer Yellow 2% plus Biocytin 2%, Molecular Probes) impaled the selected somata of brain slices immersed in PB 0.1 M under the Sylgard base of the injection chamber. Micropipettes connected to a current source (Neurodata IR-283, Intracellular Recording Amplifier, Cygnus Technology) were driven by two micromanipulators (Narishige MMO-203, three-axis and MO-81 pulse motor microdrive). Selected neurons were impaled and injected with the tracer. Lucifer Yellow (LY) (LY CH, lithium salt, L-453, Molecular Probes, 0.1% in distilled water, 1–3 nA negative current for 10–15 min in 25 ms ON/OFF cycles) was firstly injected to observe how the neuron membrane produced no tracer leaks. Then the current polarity was changed and biocytin (biocytin-*E*-biotinoyl−/−lysine, B-1592, Molecular Probes, 2–4% in distilled water, 1–3 nA positive current for 10–15 min) was injected. Finally, slices were postfixed in 4% GA in PB at 4°C.

Injected neurons were observed in either a fluorescence microscope (Zeiss AxioPlan or Olympus BX50WI) or a confocal microscope (Leica TCS SP2) to observe fluorescent marker labeling. Optionally, the Vectastain ABC immunoperoxidase kit (Vector Laboratories, Burlingame, CA) was used with the slices bearing the selected neurons to detect biocytin. After a brief study under a light microscope with camera lucida, slices were osmium-postfixed (1% osmium in 8% glucose for one hour) and resin-embedded using a flat mold (Durcupan, Fluka). The semithin sections (1–2 µm) containing selected byocytin-filled processes were photographed and selected for further immunocytochemical GABA detection and electron microscopic study.

### Intracellular Injections after Retrograde Labeling

To detect axonal projections from molecular layer neurons, attempts of retrograde tracing were done by means of tracer injections in several adjacent structures. Firstly, 100–200 nl of either 1% DiI, (1,1′dioctadecyl-3,3,3′3′-tetramethyl-indocarbocynine perchlorate, Molecular Probes) in dimethyl sulfoxide or 1% WGA-HRP, wheat germ agglutinin-horseradish peroxidase in phosphate buffer 0.1 M pH 7.4, were injected into the subiculum of 30-day-old rabbits anesthetized with an intramuscular injection of 25 mg/kg Ketamine and 0.5 mg/kg Medetomidine. Subicular injections were performed with the help of a microsyringe (Syringe 10 µl Flexifil Tapertip, World Precision Instruments, Inc.) and a stereotactic instrument (SR-5R-HR plus universal adaptor SM-15, Narishige Scientific Instrument Lab.); coordinates Postbregma 6 mm posterior, lateral 3.5–4 mm and depth 3–5 mm.

At 2 to 5 days after the injection of tracers, animals were perfused, brains extracted and slightly fixed slices obtained as previously described. The retrograde DiL labeled cells in the slices were again intracellularly injected with Lucifer yellow and biocytin to be processed and studied in the same way. WGA-HRP-labeled cells were revealed with 0.03% DAB, 0.33 µl/ml H_2_O_2_ in phosphate buffer 0.1 M pH 7.4.

### GABA Immunocytochemistry

Semithin sections from the brain slices of animals perfused with glutaraldehyde and embedded in epoxy resin for electron microscopy were used for GABA immunostaining. In short, the resin was removed from semithin sections with a mixture of 50% sodium etoxide, 30% acetone and 20% toluene, which were then washed with absolute ethylic alcohol, rehydrated and exposed overnight with a drop of primary antibody (in a wet chamber to prevent desiccation; a circle of Pap-Pen around sections prevented the antibody from dispersing) (1∶500 dilution of rabbit Anti-GABA, Sigma A2052). The secondary antibody used was goat anti-rabbit IgG FITC-conjugated, Sigma F9887 (dilution 1∶200).

Electron microscopy GABA immunostaining was performed in some ultrathin sections collected on formvar-coated single-slot nickel grids. They were treated with 1% periodic acid to etch the resin surface and 1% sodium metaperiodate to remove osmium. After pre-incubation in 10% normal goat serum, sections were incubated with a polyclonal antibody against GABA raised in rabbits (Sigma) diluted to 1∶500, followed by incubation with gold-conjugated goat anti-rabbit IgG (10 nm diameter gold particles, Sigma) diluted 1∶10. Finally, sections were counterstained with 2% uranyl acetate and lead citrate and observed with the electron microscope. Immunocytochemical controls, including the omission and substitution of the primary antibody by normal rabbit serum, followed by incubation with the remaining immunoreagents, showed no immunostaining.

### Electrophysiological Recordings

Animals (P20–21) were anesthetized deeply with halothane gas and decapitated. Brains were excised and immersed rapidly in oxygenated ice-cold (4–6°C) artificial CSF (ACSF) with sucrose (234 mM), thus replacing NaCl (117 mM) to maintain osmolarity. Hippocampal slices (400 µm thick) were cut in cold oxygenated Ringer's solution using a Vibratome S1000 (Technical Products International, O'Fallon, MO), and placed in an incubation chamber where they were maintained for ∼2 h at room temperature. For recordings, a single slice was transferred to an interface recording chamber (BSC-HT and BSC-BU; Harvard Apparatus, Holliston, MA) and perfused continuously with ACSF comprising the following (in mM): 117 NaCl, 4.7 KCl, 2.5 CaCl_2_, 1.2 MgCl_2_, 25 NaHCO_3_, 1.2 NaH_2_PO_4_, and 11 glucose. The ACSF was bubbled with carbogen gas (95%O_2_–5%CO_2_) and maintained at 30±2°C. Recorded neurons were identified by the routine procedures used in our laboratory [36]. Intracellular records from the hippocampal neurons were obtained with borosilicate glass microelectrodes (140–180 MΩ; World Precision Instruments, Sarasota, FL) filled with biocytin diluted in a 2 M potassium acetate solution [37] and connected to the head stage of an intracellular recording amplifier (VF180; Biologic, Claix, France). Micropipette tips were directed to the hippocampal ML. Only the data from the neurons that had a stable resting potential with absolute values greater than −50 mV in the absence of DC holding currents and which presented overshooting action potentials were collected for analysis. Spike amplitude potentials were measured in relation to the threshold. Neurons were stained by the intracellular injection of biocytin using positive current pulses of 0.2 nA for 6 min. Slices were fixed and cut into sections (45 µm) using a freezing microtome (HM400R; Microm, Heidelberg, Germany). Sections were revealed with the avidin-biotin-peroxidase complex (Vector Laboratories, Burlingame, CA), and 3,3'-Diaminobenzidine was used as a chromogen to visualize the biocytin complex. Sections were counterstained with cresyl violet. Neurons were reconstructed from serial sections using a camera lucida (Nikon Labophot; Nikon, Kawasaki, Japan).

## Supporting Information

Figure S1 **Nissl staining of the rabbit hippocampal molecular layers.** Eight neuronal archetypes in the hippocampal molecular layer could be identified and distinguished in animals of all different ages using Nissl staining technique (at P6, P8, P10, P12, and P14 showed), according different morphology and location of the neuronal soma. Neuron archetypes from up to down: sh- small horizontal; ip- inverted pyramidal; v- vertical; g- globoid; p- polymorphic; s- sarmentous; e- ectopic granular; lh- large horizontal. Scale bar, 25 µm.
(TIF)
