# Supplementary figures and images for: Neurons of the Dentate Molecular Layer in the Rabbit Hippocampus
Source: PLoS One. 2012 Nov 7;7(11):e48470. doi: 10.1371/journal.pone.0048470 (PMC3492497; doi:10.1371/journal.pone.0048470)

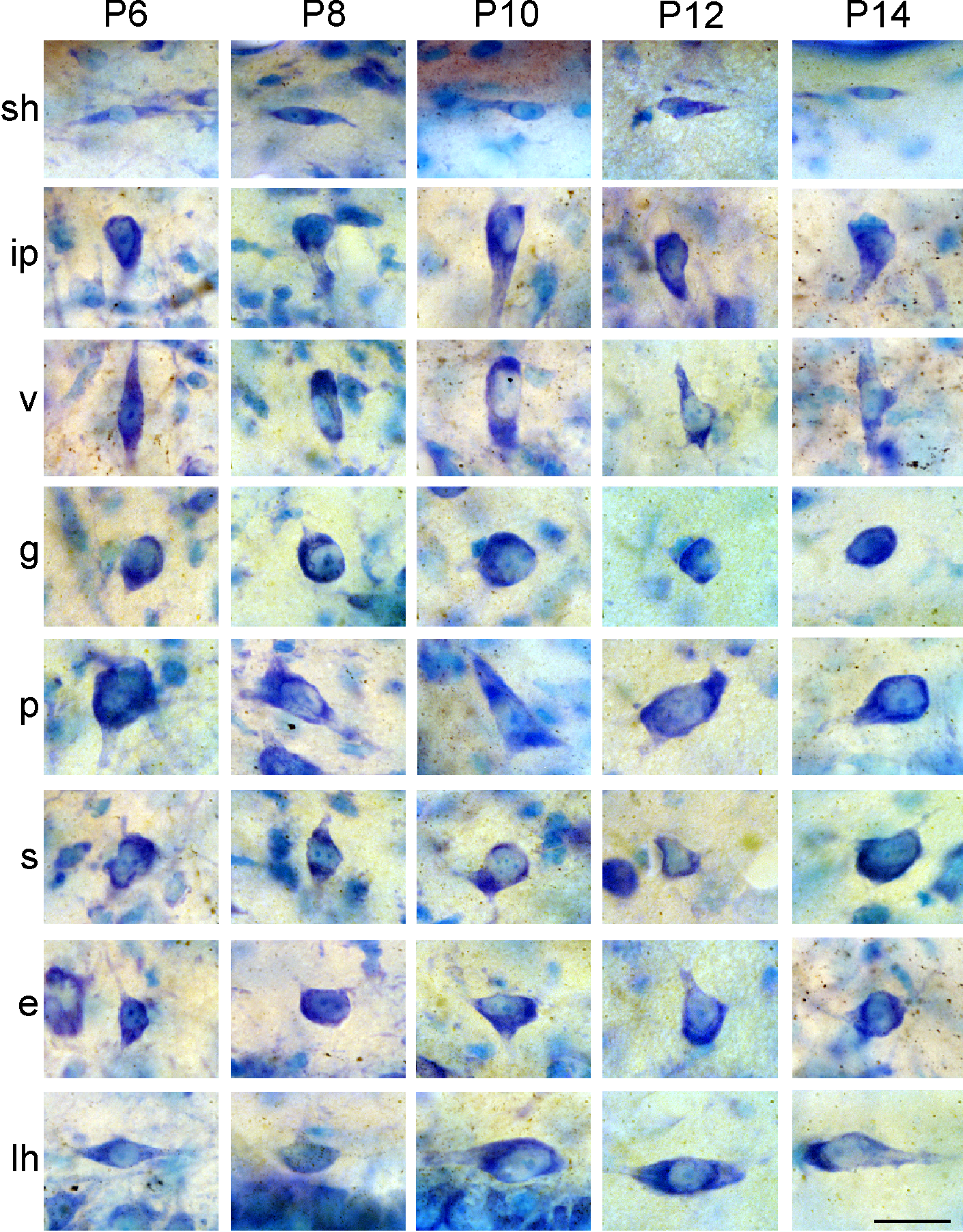

Supplement: Figure S1 — Nissl staining of the rabbit hippocampal molecular layers. Eight neuronal archetypes in the hippocampal molecular layer could be identified and distinguished in animals of all different ages using Nissl staining technique (at P6, P8, P10, P12, and P14 showed), according different morphology and location of the neuronal soma. Neuron archetypes from up to down: sh- small horizontal; ip- inverted pyramidal; v- vertical; g- globoid; p- polymorphic; s- sarmentous; e- ectopic granular; lh- large horizontal. Scale bar, 25 µm. (TIF) [file pone.0048470.s001.tif]
